# Alternative Approaches for Assessing Cassava Brown Streak Root Necrosis to Guide Resistance Breeding and Selection

**DOI:** 10.3389/fpls.2019.01461

**Published:** 2019-11-14

**Authors:** Robert Sezi Kawuki, Williams Esuma, Alfred Ozimati, Ismail Siraj Kayondo, Leah Nandudu, Marnin Wolfe

**Affiliations:** ^1^National Crops Resources Research Institute (NaCRRI), Kampala, Uganda; ^2^Section on Plant Breeding and Genetics, Cornell University, Ithaca, NY, United States

**Keywords:** breeding, cassava brown streak disease (CBSD), necrosis, resistance, virus

## Abstract

Cassava brown streak disease (CBSD) caused by the rapidly evolving cassava brown streak viruses (CBSVs), causes immense yield losses to the cassava value chain in eastern and southern Africa. Western Africa, another region that heavily depends on cassava is under eminent threat from CBSD. Resistance breeding is the best practical solution. However, complexities associated with CBSD resistance screening i.e., variable root sampling units, limit systematic attainment of genetic progress. Accordingly, we compared efficiency of five CBSD root necrosis assessment methods to guide selection: cassava brown streak disease root incidence (CBSDRi), cassava brown streak disease root severity (CBSDRs), cassava brown streak disease root severity computed as harmonic mean (CBSD-Harmonic), proportion-based root necrosis index (CBSD-proportion), and standardized root necrosis index (CBSD-standardized). The indexes (CBSD-proportion and CBSD-standardized) correct for variable sample size. We analyzed CBSD root necrosis data of 256 clones evaluated across 12 environments. Higher and variable standard errors were associated with root severity score 1 (no CBSD root necrosis). Lowest and highest plot-based heritability were respectively registered for CBSD-standardized (0.22) and CBSD-proportion (0.71). CBSDRs was only positively correlated with CBSDRi (r = 0.92) and CBSD-Harmonic (r = 0.97). Using best linear unbiased predictions (BLUPs), we ranked the top 15 CBSD resistant clones; only one clone (UG130014) featured in all the five assessment methods; two clones (UG130006 and UG120156) featured in four (CBSD-Harmonic, CBSDRi, CBSDRs, and CBSD-standardized); and five clones (UG120180, UG120063, UG130002, UG130033, and UG120183) featured in three methods (CBSD-Harmonic, CBSDRi, and CBSDRs). Influence of sample size was also quantified by sub-setting and analyzing CBSDRs data to have plots with at least 40 or 30 roots. Data stabilization was evident in plots with 30 roots. The significant influence of root sample sizes on overall ranking of clones, justifies the use of CBSD root necrosis indexes in early selection stages i.e., seedling and/or clonal trials, that are often characterized by high variations in roots assessed per plot. It is expected that this information will provide a foundation for harmonizing and/or optimizing on-going and future CBSD resistance breeding efforts.

## Background

Cassava’s (*Manihot esculenta* Crantz) clonal nature, tolerance to marginal soils and ability to provide diverse food and non-food uses have elevated its importance in several countries within southern, eastern and western Africa. On aggregate, these regions produce and consume more than 54% of the world’s cassava (FAOSTAT, 2019), essentially highlighting cassava’s significance in Africa. Since cassava’s introduction on the African continent *via* the western and eastern coastlines between the 15^th^ and 17^th^ century (Olsen and Schaal, 2002; Olsen, 2004), the crop has suffered from several overlapping stresses, whose severity varies across countries (Legg et al., 2014).

Cassava brown streak disease (CBSD) caused by two ssRNA virus species, Uganda cassava brown streak virus (UCBSV) and cassava brown streak virus (CBSV), have both viruses, been restricted to southern and eastern Africa for the past 90 years (Ndunguru et al., 2015; Alicai et al., 2016; Munganyinka et al., 2018), with no presence in Latin America, the center of origin of cassava and/or in west Africa. The negative impact of CBSD on both the quality and quantity of marketable cassava roots makes it an acute factor limiting aspirations of cassava commercialization in eastern and southern Africa. In fact, CBSD-affected roots have been reported to show reductions of 30% for amylose content, 50% for amylopectin content and 15% for total starch content (Nuwamanya et al., 2015). It is for these reasons that cassava breeding efforts in most southern and eastern African countries currently prioritize CBSD resistance as a major objective.

Typically, CBSD symptoms appear as characteristic chlorotic leaf symptoms along the major veins, pronounced brown streak lesions on stems and necrotic corky roots (Hillocks and Jennings 2003; Munganyinka et al., 2018). The starch-bearing roots, which by far are the most economic part of cassava, are worst hit by CBSD. Unfortunately, poor phenotypic correlations exist between above-ground (foliar and stems) and the below ground symptoms (Kaweesi et al., 2014; Kawuki et al., 2016; Okul Valentor et al., 2018), a phenomenon that complicates evaluation and selection for CBSD resistance. It’s partly for these reasons that the manuscript focused on CBSD root necrosis assessment.

In practice however, resistance breeding necessitates having disease categorization methods that can reliably confirm resistance or susceptibility levels of evaluated individuals. For the case of CBSD, resistance or tolerance has been defined using incidence and severity scores of foliar and/or root symptoms (Rwegasira and Rey, 2012; Okul Valentor et al., 2018). Other studies have combined both symptoms and virus titre to categorize genotype response (Mohammed et al., 2012; Kaweesi et al., 2014; Sheat et al., 2019). Briefly, CBSD incidence is defined as the proportion of plants or roots of a clone expressing disease symptoms in a given plot, while severity is the degree of CBSD symptoms. This assessment can be done on individual plants or on a plot basis (pooled plants). The two CBSD root necrosis assessment methods highlighted above (incidence and severity), are associated with two major biases, which this study proposed to highlight and address.

Firstly, unlike the straightforward case of foliar incidence (proportion of plants exhibiting CBSD leaf and/or stem symptoms), CBSD root necrosis incidence assessment is much more challenging. From experience, we observe that different genotypes (or clones) exhibit varying frequencies of root severity scores per plant; moreover, the number of roots assessed per plant and/or per plot also varies considerably. These have implications on the overall inference of resistance/tolerance levels of such genotypes.

The second point of contention arises with methodology for assessing root severity using the standard 1–5 scale (Gondwe et al., 2003), where 1 = no necrosis, 2 = mild necrotic lesions (1–10%), 3 = pronounced necrotic lesions (11–25%), 4 = severe necrotic lesions (26–50%) and 5 = very severe necrotic lesions (> 50%). It suffices to note that a root that is assigned a score of 1 is economically more valuable than that scoring 2, 3, 4, or 5. This intuition (differences in roots assigned different scores per plot and/or per plant) has never been used for CBSD root necrosis assessment.

Thus, herein we compared the traditional CBSD root necrosis assessment methods (severity and incidences) with CBSD root necrosis indices, which accounts for the variable number of roots assessed per plot or the economic value of roots assessed based on the 1–5 scale. For this purpose, we analyzed CBSD root necrosis data of 256 cassava clones that were established in three locations for four consecutive seasons (2015A, 2015B, 2016A, and 2016B). Data associated with all these clones and trial sites has been deposited at https://www.cassavabase.org/breeders/trials/. Specifically, we addressed the following questions. a) To what extent do CBSD root necrosis assessment methods namely: cassava brown streak disease root incidence (CBSDRi), CBSD root severity (CBSDRs), CBSD root severity computed as harmonic mean (CBSD-Harmonic), proportion-based CBSD root necrosis index (CBSD-proportion), and standardized root necrosis index (CBSD-standardized), accurately rank cassava clones in their response to CBSD? b) To what extent does root sample size bias CBSD root necrosis assessment?

## Methodology

### Genetic Materials

A total of 256 diverse cassava clones were used. These clones were generated in 2010 from 49 progenitors that were collectively sourced from a) International Institute of Tropical Agriculture (IITA), b) International Centre for Tropical Agriculture (CIAT) and c) Tanzania. These clones were part of a panel of 429 clones that constituted a cycle-0 population described in previous studies by Ozimati et al. (2018). This population segregated for CBSD resistance, with pedigree information available at https://www.cassavabase.org/breeders/trials/. 

### Field Evaluations

Field trials were conducted at three locations in Uganda: Namulonge (central region), Serere (eastern region) and Ngetta (northern region). The study sites receive bimodal rainfall patterns with first rains (A) commencing in April and ending in June, while second rains (B) fall between September and November. At each location, trials were planted twice per year, following the two rain seasons (April–June and September–November). Trials planted during the first rains (A), commencing in April, were harvested at 12 months after planting (MAP), which coincided with the month of April of the following year. Similarly, trials planted during the second rains (B) of the same year, commencing in September, were harvested at 12 MAP, which coincided with the month of September of the following year. Thus, two plantings were made per year: two plantings A and B in 2015 and two plantings A and B in 2016. Altogether, four field evaluations per site (2015A, 2015B, 2016A, and 2016B) were undertaken.

At each site, trials were laid out in an augmented design comprising five checks (UG110008, UG110014, UG110015, UG110016, and UG110017); each check was replicated five or six times and was represented in each block. The check clones consistently display susceptible (UG110008, UG110015, and UG110016) and tolerant (UG110014 and UG110017) reactions to CBSD root necrosis (Kawuki et al., 2016; Okul Valentor et al., 2018). Each test clone was represented by a single row of 10 plants. Plant spacing of 1 m × 1 m was adopted between and within rows, while blocks were separated by 2 m alleys. Stems cuttings of a CBSD susceptible clone TME 204 (syn. UG110016) were sourced from farmer’s fields that had ≥ 80% CBSD incidence and mean CBSD severity of ≥ 4 for shoot and/or roots. Consequently, these cuttings were planted along the boundaries of the main evaluations plots and used as spreaders to effectively augment CBSD pressure.

Thus, CBSV infection on the test clones was natural and was greatly aided by the presence of high whitefly, *Bemisia tabaci* populations (Omongo et al., 2012; Mugerwa et al., 2018) and high CBSD inoculum (characterized by both CBSV and UCBSV virus species) across the three evaluations sites (Mukiibi et al., 2019). No virus titre data were collected from these trials, as it was logistically inappropriate, and not the focus of this study. All trials were kept weed-free by regular weeding. At harvest, which coincided with 12 months after planting (MAP), all plants/row were uprooted and all roots individually assessed using the 1–5 scale as described earlier (Kaweesi et al., 2014). In addition, root dry matter content (DMC) was measured using a sample of 2 to 5 kg of roots sampled per plot; these were weighed in air and in water to enable computation of specific gravity, which was subsequently used to estimate DMC, as described by Kawano et al. (1987).

### Data Analyses

Using data generated from the 12 trials comprising of three locations (Namulonge, Serere, and Ngetta) and four seasons (2015A, 2015B, 2016A, and 2016B), the following analyses were undertaken. First, CBSD root incidences were computed on plot basis, as a proportion of infected roots to total number of roots harvested per plot, and multiplied by 100 (CBSDRi). Secondly, average CBSD root necrosis severity (including scores of 1 in order to capture all plot variability) was computed on plot basis, as average root necrosis across all roots assessed per plot (CBSDRs). Thirdly, we computed harmonic means for CBSD root necrosis severity on a plot basis using all roots scored per plot (CBSD-Harmonic).

To account for the variable number of roots assessed per plot (clone) and the varying economic values of roots scored as 1, 2, 3, 4, or 5, we computed two indices. First, the proportion-based CBSD root necrosis index (CBSD-proportion) that was computed following four steps sequentially: step 1) we counted the number of roots that were assigned to each root necrosis category i.e., roots that were respectively assigned scores of 1, 2, 3, 4, or 5 per plot; step 2) we computed proportions (*p*) for each root necrosis category as $\frac{number\;of\;roots\;assigned\;to\;necrosis\;category}{Total\;number\;of\;roots\;scored\;per\;plot}$ and thus generated five proportions per plot; step 3) for each proportion computed, we multiplied by the respective index weights (*x*) assigned to each root necrosis category. Specifically, we assigned a positive value (+1) for root necrosis score 1 (no root necrosis); negative value (−0.9) for root necrosis score 2 (mild necrotic lesions, 1–10%); negative value (−0.75) for root necrosis score 3 (pronounced necrotic lesions, 11–25%); negative value (−0.5) for root necrosis score 4 (severe necrotic lesions, 26–50%); and negative value (−0.25) for root necrosis score 5 (very severe necrotic lesions >50%). Ideally a positive value is assigned for what is desirable and higher negative values for what is undesirable.

For step 4) we summed up products (*px*) generated in step 3 across the five root necrosis categories to get a single value per clone. For this we used the formula $\overset{5}{\underset{i\;=\;1}{\sum }}px$ where *p* is the computed proportion and *x* is the assigned index weight. In principle, positive values for CBSD-proportion depict CBSD resistance, while negative values are indicative of CBSD susceptibility. The second index was based on standardized CBSD root necrosis (CBSD-standardized). For this index, we opted to standardize to correct for the variable number of roots assessed per plot. For this we used the formula $\;\frac{x-\;\mu \;}{\delta }$,where *x* = number of roots assigned to a specific necrosis category, *µ* and *δ* are respectively, the mean and standard deviation of a specific necrosis category across all plots evaluated. CBSD-standardized index was computed using the four sequential steps described above, except that in step 2, we standardized the root counts for each root necrosis category. Again, positive values depict CBSD resistance, while negative values depict CBSD susceptibility.

Combined datasets were subjected to correlations analysis using the *cor* function in R. In addition, mixed model analyses were conducted using the *lmer* function in R to estimate variances associated with each of the five CBSD root necrosis assessment methods (traits), namely CBSDRi, CBSDRs, CBSD-Harmonic, CBSD-proportion, and CBSD-standardized. For these analyses, clones, blocks nested in sites, year/clone interactions, and site/clones’ interactions were considered random effects, while seasons were considered as fixed effects. Broad-sense heritability for each of the five CBSD root necrosis traits were computed. Furthermore, best linear unbiased predictions (BLUPs) were computed for each clone, the main random effect; these provided a basis for comparing ranks for clones based on the five CBSD root necrosis assessment traits. Finally, we computed the standard errors associated with each of the 1–5-point severity scale.

To examine the extent to which root sample size influenced CBSD root necrosis assessment, the combined data was randomly split into seven subsets (groups) comprising of clones with varying number of roots assessed per plot. For simplicity, mindful that the trial had mean root number of 23.2, we categorized the groups as follows: group 1 (11–20 roots); group 2 (21–30 roots); group 3 (31–40 roots); group 4 (41–50 roots); group 5 (51–60 roots); group 6 (61–70 roots), and group 7 (71–95 roots). Analysis of variance was conducted for the five CBSD root necrosis traits to establish whether or not significant differences existed between the groups. The *lsmean* function in R was used to generate least square means and contrasts to enable comparisons among groups. For this analysis, we fitted a linear model comprising grouping, sites, year and clone as factors.

To further assess the influence of per-plot root sample size, we subset the data into two categories: one with all plots having ≥ 40 roots and the other, for all plots with ≥ 30 roots; clones overlapped between the subsets. For this analysis, we focused on CBSDRs, CBSD-Harmonic, CBSD-proportion, and CBSD-standardized. We then sampled between 5, 10, 20, 30, or 40 roots from each plot at random and computed the plot-means. We generated five random samples of roots per sample size, per data subset. For each replicate dataset, we fitted the same mixed model described above, again using *lmer*. For each analysis, we extracted the following model summary statistics: variance components for clone (Vg) and residual (Ve), broad-sense heritability (H^2^ = Vg/(Vg+Ve), and the number of outliers, with outliers defined as having a standardized residual value of >|3.3|. Ideally, this analysis was undertaken to inform on the minimum number of roots to sample per plot, above which statistical estimates stabilize.

## Results

### Variation, Heritabilities and Correlations Among CBSD Root Necrosis Assessment Traits

We compared five CBSD root necrosis traits namely: CBSDRi, CBSDRs, CBSD-Harmonic, CBSD-proportion, and CBSD-standardized. This comparison was based on 1,354 phenotypic data points collected on 256 clones across 12 environments. Number of roots assessed per plot ranged from 0 to 96, with a mean of 23.1; these roots were harvested from plots having between 5 to 10 plants. Standard deviations (SD) associated with each of the five-grade CBSD root severity scores were: 16.1, 4.02, 3.08, 2.23, and 5.91, respectively for assessment scores 1, 2, 3, 4, and 5 (**Table 1**).

**Table 1:** Summary statistics and CBSD root necrosis scale and assessment methods.

Summary Statistic	Root Count	CBSDRi	*CBSDRs*	CBSD-Harmonic	CBSD-Proportion	CBSD-Standardized	Score 1	Score 2	Score 3	Score 4	Score 5
Mean	23.18	37.20	1.91	1.67	0.33	−0.13	16.14	2.72	1.85	1.07	2.37
Standard Error	0.49	0.99	0.03	0.03	0.02	0.07	0.44	0.11	0.08	0.06	0.16
Standard Deviation	18.02	36.37	1.16	1.05	0.79	2.39	16.18	4.02	3.09	2.23	5.92
Sample Variance	324.80	1,322.84	1.35	1.10	0.63	5.71	261.95	16.19	9.53	4.99	35.04
Minimum value	0.00	0.00	1.00	1.00	−8.25	−19.72	0.00	0.00	0.00	0.00	0.00
Maximum Value	96.00	100.00	5.00	5.00	2.00	6.12	90.00	32.00	26.00	20.00	55.00
H^2^		0.45	0.49	0.45	0.71	0.23					

CBSDRi, cassava brown streak disease root incidence; CBSDRs, cassava brown streak disease root severity; CBSD-Harmonic, cassava brown streak disease root severity computed as harmonic mean; CBSD-proportion, proportion-based root necrosis index; CBSD-standardized, standardized root necrosis index. Score 1 = no necrosis; Score 2 = ≤ 5% necrotic; Score 3, 6-10% necrotic; Score 4, 11-25% necrotic and mild root constriction; and Score 5 = > 25% necrotic and severe root constriction. H^2^ = broad-sense heritability.

**Figure 1:**
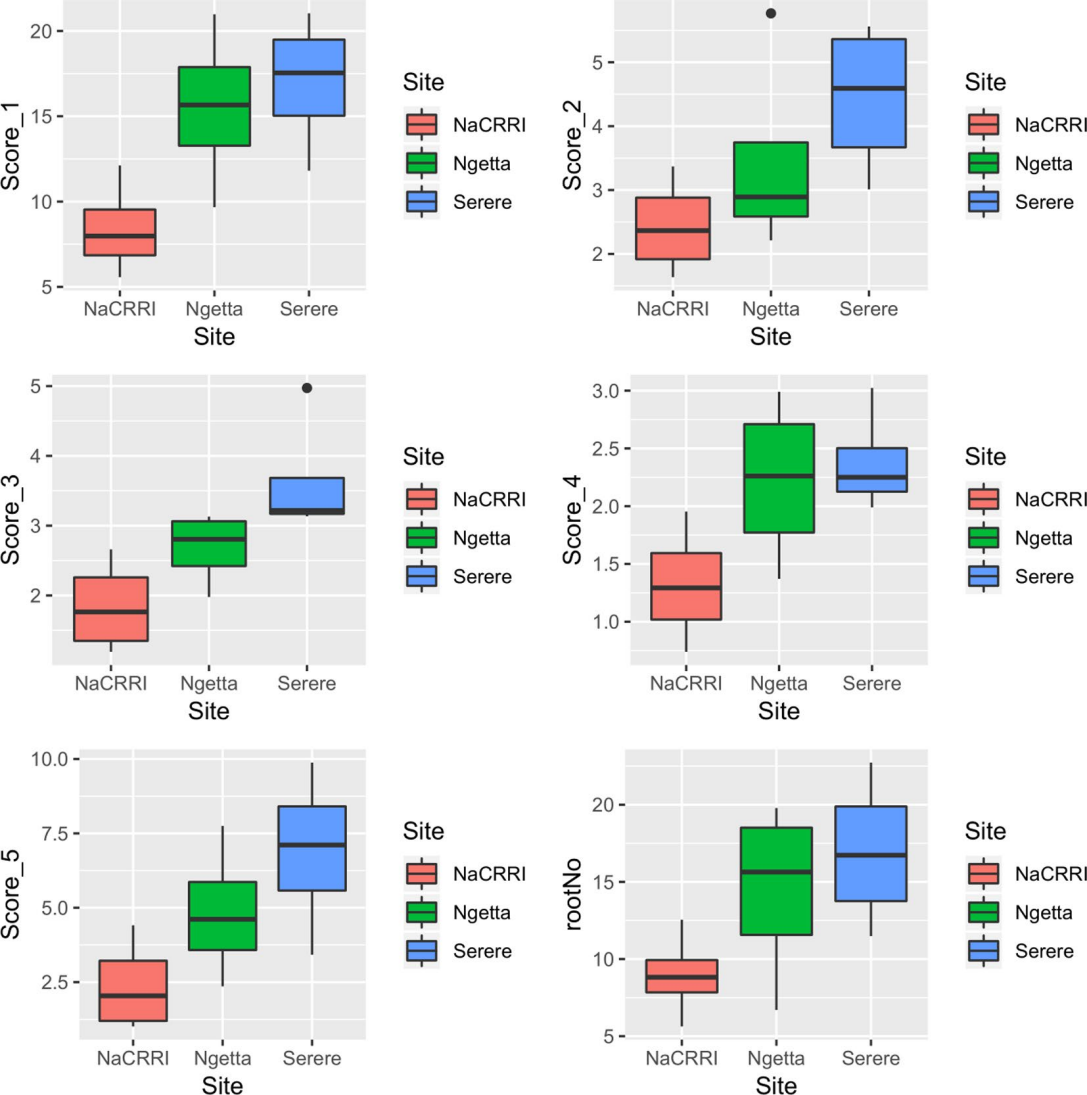
Standard errors associated with CBSD root necrosis severity scores across three sites: Namulonge (central Uganda), Ngetta (northern Uganda) and Serere (eastern Uganda). Score 1 = no necrosis; Score 2 = ≤ 5% necrotic; Score 3 = 6–10% necrotic; Score 4 = 11–25% necrotic and mild root constriction; and Score 5 = 25% necrotic and severe root constriction. RootNo = refers to number of roots sampled per plot.

CBSDRi ranged from 0 to 100% with an average score of 37.2% and SD of 36.3. Both CBSDRs and CBSD-Harmonic had respective means of 1.9 and 1.67, with respective SD of 1.16 and 1.04 (**Table 1**). CBSD-proportion index ranged from -8.2 to 2, with a SD of 0.79, while CBSD-standardized ranged from −19.7 to 6.11, with a SD of 2.33 (**Table 1**). For the 1–5 CBSD root necrosis severity scale, highest standard errors were consistently associated with severity score 1; other scale levels (particularly 2, 3, and 4) were associated with lower standard errors (**Figure 1**). Indeed, out of the 1,354 data points (evaluated plots), 152 plots had no roots that scored one, 487 plots had between 1 to 10 roots that scored one, 312 plots had between 11 to 20 roots that scored one, 150 plots had between 30–45 roots that scored one, 13 plots had between 70–80 roots that scored one; many other variations were observed (**Supplementary Table 1**). Overall, CBSD root necrosis data from Namulonge (NaCRRI) was associated with lower standard errors compared to data generated from the other two locations, Ngetta and Serere (**Figure 1**).

**Figure 2:**
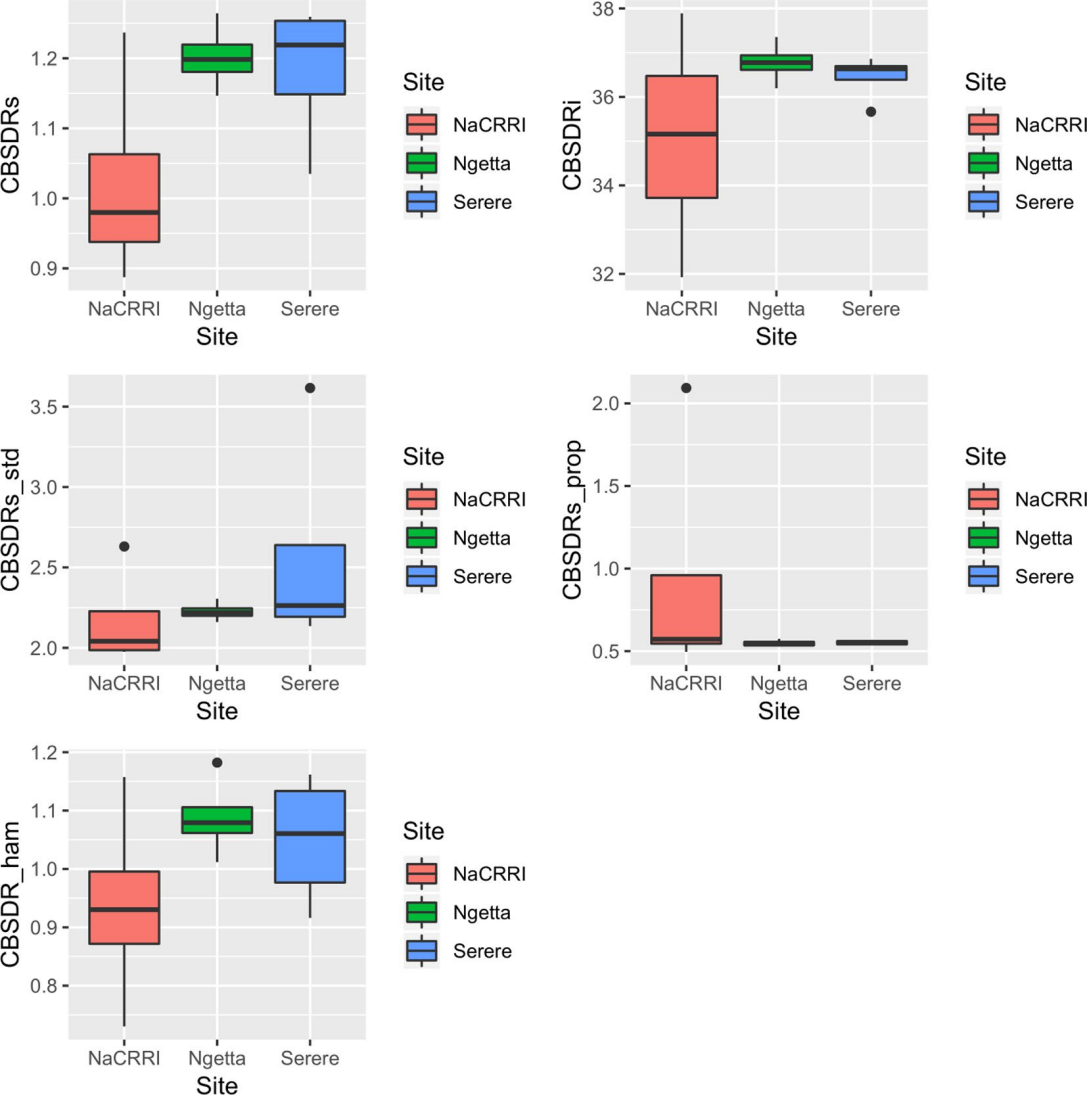
Standard errors associated with CBSD root necrosis assessment traits across three sites: Namulonge (central Uganda), Ngetta (northern Uganda) and Serere (eastern Uganda). CBSDRi cassava brown streak disease root incidence; CBSDRs, cassava brown streak disease root severity; CBSD-Harmonic, cassava brown streak disease root severity computed as harmonic mean; CBSD-proportion, proportion-based root necrosis index; CBSD-standardized, standardized root necrosis index.

**Figure 3:**
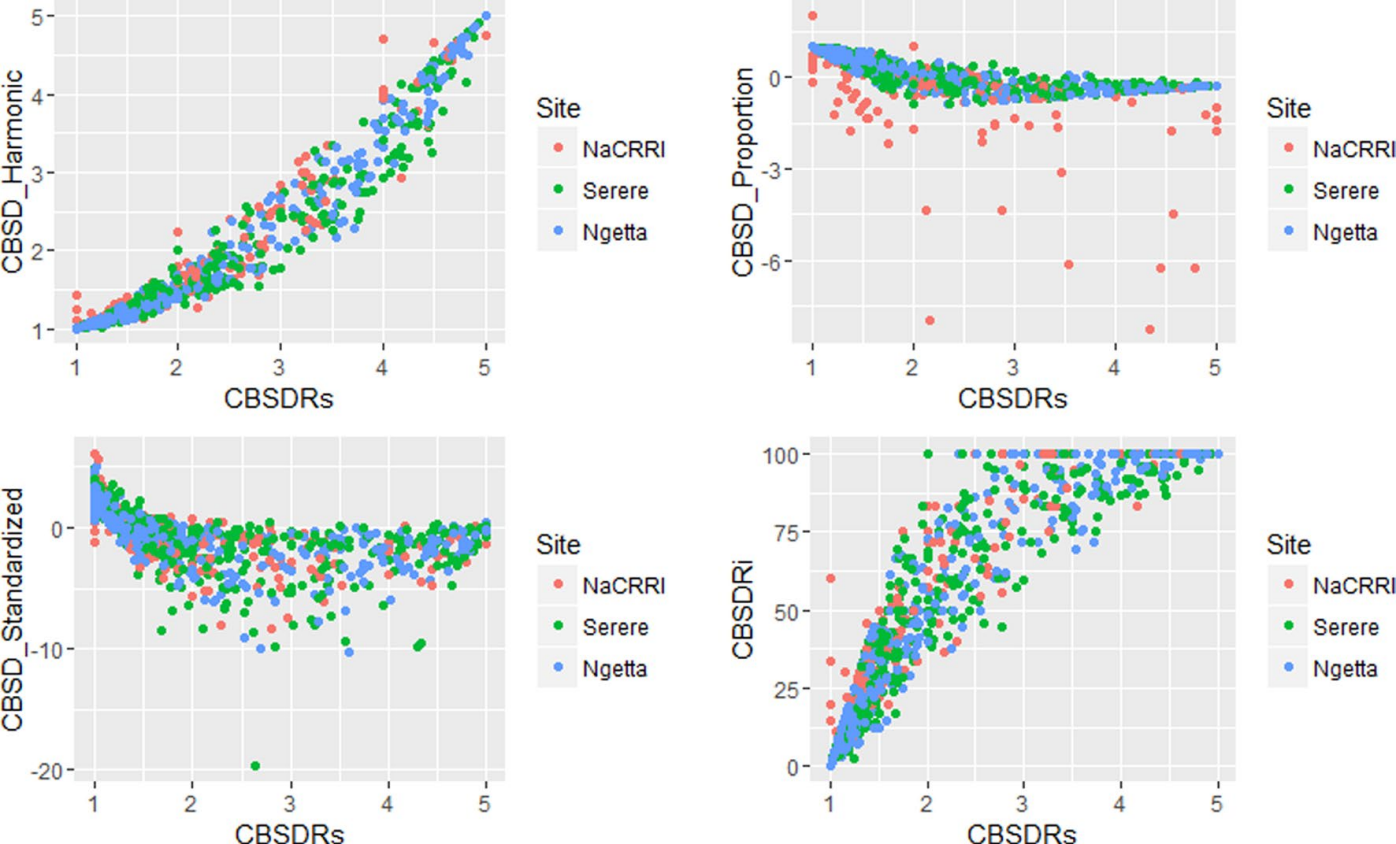
Correlations amongst the CBSD root necrosis assessment traits across three sites: Namulonge (central Uganda), Ngetta (northern Uganda) and Serere (eastern Uganda). CBSDRi, cassava brown streak disease root incidence; CBSDRs, cassava brown streak disease root severity; CBSD-Harmonic, cassava brown streak disease root severity computed as harmonic mean; CBSD-proportion, proportion-based root necrosis index; CBSD-standardized, standardized root necrosis index.

Amongst the five CBSD root necrosis traits, highest standard errors and their respective variations were associated with CBSDRi (**Table 1**; **Figure 2**). Again, data from Namulonge were associated with lower standard errors as compared to other sites (**Figure 2**). We observed the lowest plot-based heritability for CBSD-standardized (H^2^ = 0.22) and the highest for CBSD-proportion (0.71); both CBSDRi and CBSD-Harmonic had heritability (H^2^) of 0.44 (**Table 1**). Phenotypic correlations between traits varied markedly (**Table 2**; **Figure 3**). For example, CBSDRs was only positively correlated with CBSDRi (r = 0.92) and CBSD-Harmonic (r = 0.97). On the other hand, negative correlations with CBSDRs were observed for CBSD-standardized (r = −0.56) and CBSD-proportion (r = −0.66). CBSD-Harmonic was negatively correlated with CBSD-standardized (r = −0.47) and CBSD-proportion (r = −0.61). Weak negative correlations (r ≤ −0.13) persisted between agronomic traits (root weight/plant and root dry matter) and CBSD root necrosis-related traits notably CBSDRi, CBSDRs, and CBSD-Harmonic (**Table 2**). On the contrary, weak positive correlations (r ≤ 0.17) persisted between agronomic traits (root weight/plant and root dry matter) and CBSD indexes (CBSD-proportion and CBSD-standardized).

**Table 2:** Correlation among CBSD root necrosis traits.

Traits	CBSDRi	CBSDRs	CBSD-Harmonic	CBSD-Standardized	CBSD-Proportion	Root Weight	DMC
CBSDRi	1.00	0.92	0.87	−0.73	− 0.77	− 0.19	− 0.28
CBSDRs	0.92	1.00	0.97	− 0.57	− 0.67	− 0.19	− 0.31
CBSD-Harmonic	0.87	0.97	1.00	− 0.47	− 0.62	− 0.20	− 0.30
CBSD-Standardized	− 0.73	− 0.57	− 0.47	1.00	0.61	0.06	0.17
CBSD-Proportion	− 0.77	− 0.67	− 0.62	0.61	1.00	0.17	0.14
Root weight	− 0.19	− 0.19	− 0.20	0.06	0.17	1.00	− 0.02
DMC	− 0.28	− 0.31	− 0.30	0.17	0.14	− 0.02	1.00

CBSDRi, cassava brown streak disease root incidence; CBSDRs, cassava brown streak disease root severity; CBSD-Harmonic, cassava brown streak disease root severity computed as harmonic mean; CBSD-proportion, proportion-based root necrosis index; CBSD-standardized, standardized root necrosis index. Root weight, root weight measured per plant; DMC, root dry matter content.

### Ranking of Cassava Clones Based on CBSD Root Necrosis Traits

We ranked the top and worst 15 clones based on BLUPs computed from each of the CBSD root necrosis-related traits (**Table 3**). Both CBSDRi and CBSDRs ranked UG120024, as the most CBSD resistant clone, while CBSD-Harmonic and CBSD-standardized, ranked UG120136 as the most CBSD resistant clone (**Table 3**). Clone UG130013 was only ranked among the top 15 by CBSD-proportion index (**Table 3**). On the other hand, three necrosis assessment methods (CBSDRi, CBSDRs, and CBSD-Harmonic) consistently ranked UG110016 as the most CBSD susceptible clone (**Table 3**).

**Table 3:** Ranking of the top 15 and last 15 clones based on best linear unbiased predictions (BLUPs).

Clone	CBSD-Harmonic	Clone	CBSDRi	Clone	CBSDRs	Clone	CBSD-Proportion	Clone	CBSD-Standardized
**Top 15**									
UG120136	− 0.461	UG120024	− 28.21	UG120024	− 0.65	UG130013	1.39	UG120136	1.90
UG130004	− 0.455	UG130014	− 26.95	UG120156	− 0.64	UG130123	0.87	UG130010	1.72
UG130014	− 0.454	UG120136	− 26.52	UG130004	− 0.64	UG130029	0.87	UG130004	1.46
UG120024	− 0.454	UG120156	− 26.47	UG130014	− 0.64	UG120198	0.85	UG130006	1.40
UG130006	− 0.447	UG130004	− 26.01	UG120136	− 0.64	UG120071	0.82	UG120186	1.37
UG120156	− 0.446	UG130033	− 25.42	UG130006	− 0.62	UG120127	0.81	UG130002	1.31
UG120180	− 0.442	UG120031	− 24.74	UG120063	− 0.61	UG120128	0.79	UG130014	1.27
UG120063	− 0.440	UG120063	− 24.73	UG110023	− 0.61	UG120183	0.78	UG120156	1.26
UG130002	− 0.440	UG130002	− 24.49	UG130002	− 0.60	UG130089	0.78	UG120135	1.22
UG130033	− 0.435	UG120180	− 24.35	UG120180	− 0.60	UG130014	0.75	UG120072	1.22
UG120031	− 0.430	UG130089	− 24.24	UG130033	− 0.60	UG120194	0.75	UG120174	1.21
UG120183	− 0.427	UG130006	− 24.16	UG120046	− 0.59	UG120182	0.75	UG120001	1.18
UG110023	− 0.426	UG130010	− 23.28	UG120183	− 0.58	UG120024	0.71	UG130107	1.16
UG120174	− 0.426	UG120046	− 23.12	UG130107	− 0.58	UG120002	0.67	UG120181	1.12
UG130010	− 0.421	UG130107	− 23.09	UG130089	− 0.57	UG120032	0.66	UG130016	1.09
**Last 15**									
UG130097	1.115	UG120291	39.51	UG120286	1.37	UG110008	− 1.04	UG120131	− 1.40
UG120221	1.191	UG120221	40.81	UG130097	1.38	UG120288	− 1.08	UG120292	− 1.40
UG120295	1.240	UG120131	41.34	UG110032	1.42	UG120291	− 1.08	UG130001	− 1.43
UG120220	1.253	UG120215	41.84	UG120215	1.46	UG120212	− 1.24	UG120154	− 1.43
UG120291	1.253	UG120306	41.87	UG120277	1.46	UG130115	− 1.28	UG120161	− 1.53
UG120215	1.340	UG130097	41.93	UG120295	1.46	UG120095	− 1.40	UG120267	− 1.68
UG120161	1.362	UG120201	42.22	UG120161	1.55	UG120135	− 1.44	UG120170	− 1.69
UG120277	1.419	UG120154	42.67	UG120271	1.56	UG130039	− 1.46	UG130097	− 1.81
UG120212	1.447	UG120286	45.14	UG120221	1.59	UG120079	− 1.51	UG110008	− 1.93
UG120271	1.535	UG120212	45.33	UG120078	1.60	UG120252	− 1.67	UG120146	− 1.94
UG120078	1.535	UG120161	47.03	UG120212	1.68	UG120092	− 3.11	UG120306	− 1.98
UG110008	1.836	UG120288	47.97	UG120202	1.96	UG120132	− 3.21	UG120247	− 2.16
UG120202	1.885	UG110008	51.55	UG110008	2.01	UG120172	− 4.40	UG120268	− 2.18
UG120288	1.951	UG120202	51.88	UG120288	2.02	UG120277	− 4.50	UG120201	− 2.67
UG110016	2.218	UG110016	54.20	UG110016	2.33	UG130078	− 5.87	UG120221	− 2.68

CBSDRi, cassava brown streak disease root incidence; CBSDRs, cassava brown streak disease root severity; CBSD-Harmonic, cassava brown streak disease root severity computed as harmonic mean; CBSD-proportion, proportion-based root necrosis index; CBSD-standardized, standardized root necrosis index.

It also suffices to note that among the top 15 CBSD resistant and/or tolerant clones, only one clone (UG130014) featured in each of the five CBSD root necrosis assessment traits; two clones (UG130006 and UG120156) featured in four (CBSD-Harmonic, CBSDRi, CBSDRs, and CBSD-standardized); and five clones (UG120180, UG120063, UG130002, UG130033, and UG120183) featured with three (CBSD-Harmonic, CBSDRi, and CBSDRs). Highly susceptible clones were readily discernible by the five CBSD root necrosis-related traits (**Table 3**). For example, clones like UG110008, UG110016, UG120288, UG120212, UG120221, and UG130097 were consistently identified as highly susceptible by at least four CBSD root necrosis assessment methods (**Table 3**).

### Influence of Samples Sizes on CBSD Necrosis Assessment

**Figure 4:**
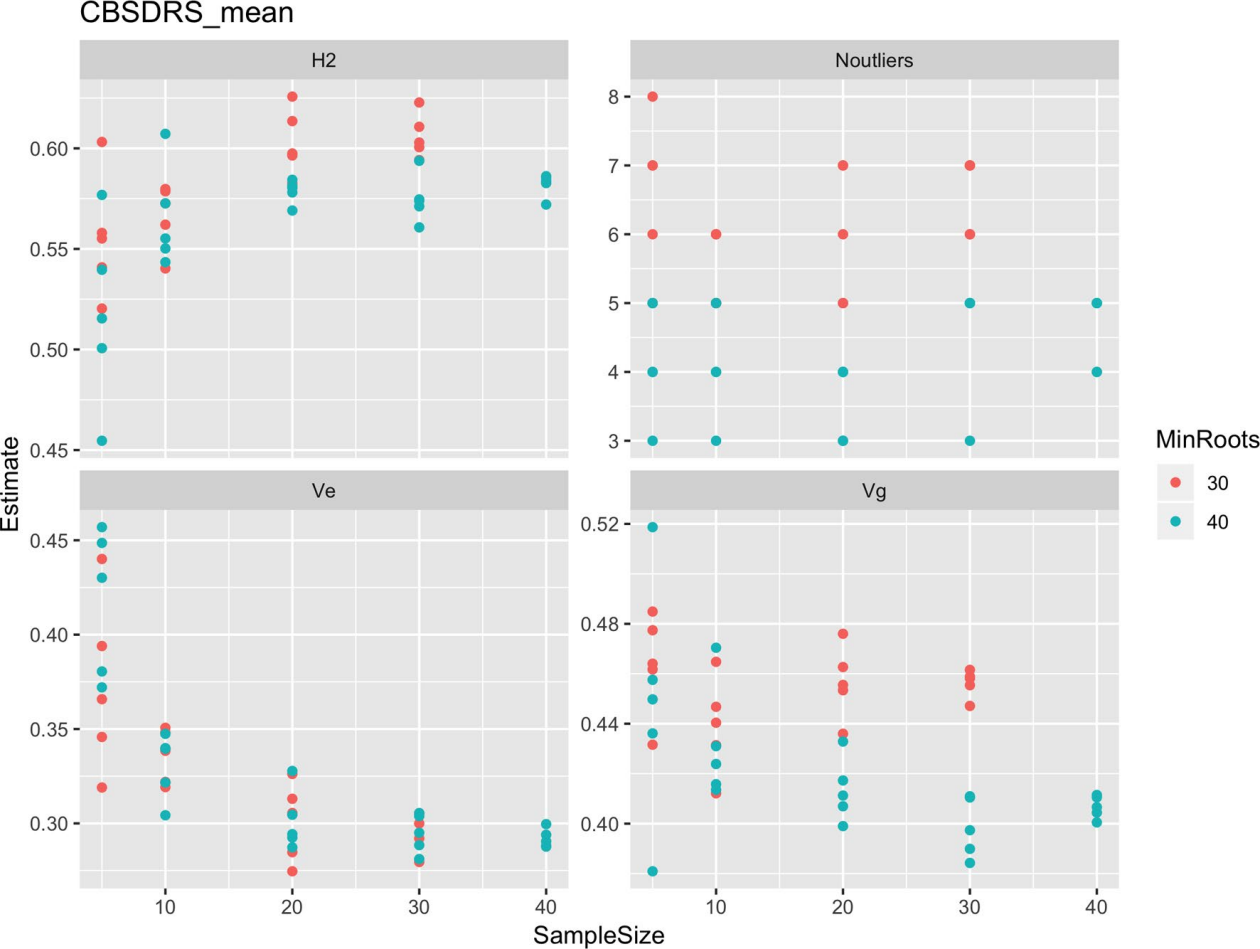
Influence of root sample sizes on CBSD mean root necrosis severity. H^2^, broad-sense heritability; Ve, error variance component; Vg, genetic variance component; Noutliers, number of outliers. Analysis based on resampling of 5, 10, 20, 30, or 40 roots from each plot at random.

**Figure 5:**
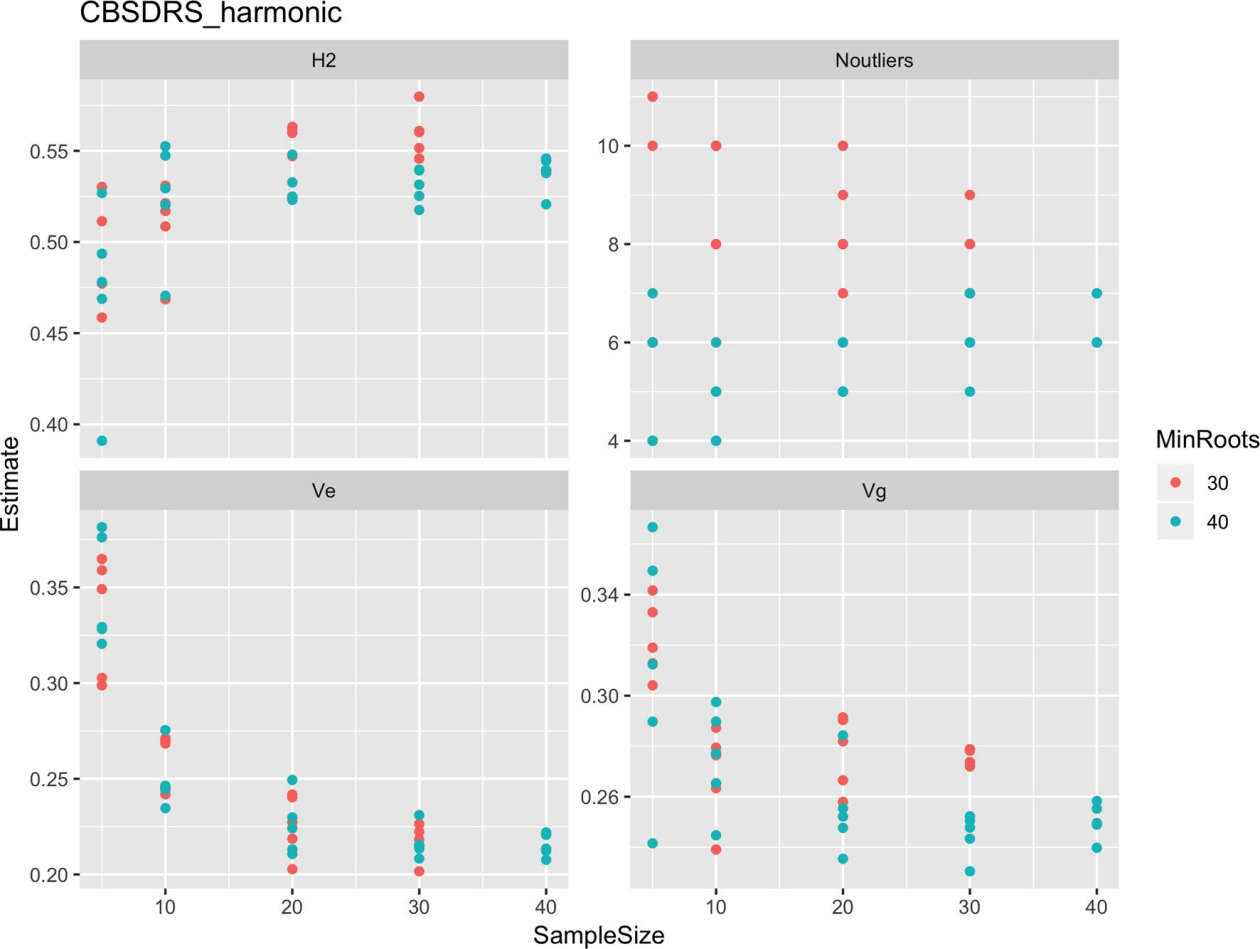
Influence of root sample sizes on cassava brown streak disease root severity computed as harmonic mean (CBSD-Harmonic). H^2^, broad-sense heritability; Ve, error variance component; Vg, genetic variance component; Noutliers, number of outliers. Analysis based on resampling of 5, 10, 20, 30, or 40 roots from each plot at random.

**Figure 6:**
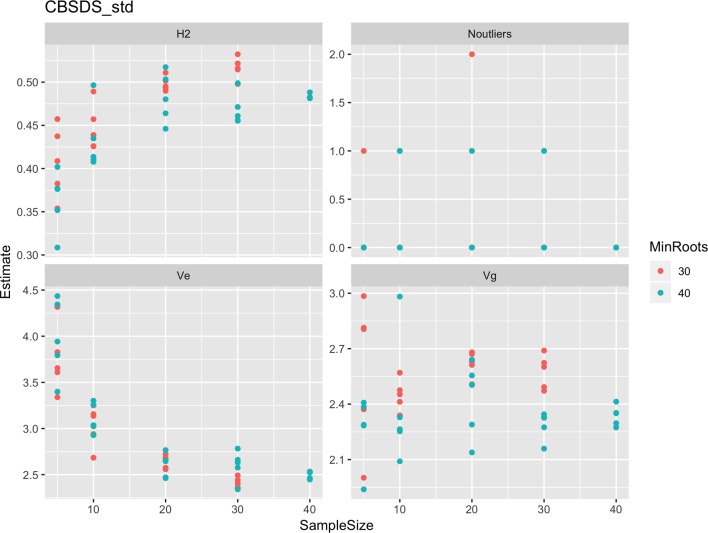
Influence of root sample sizes on standardized root necrosis index (CBSD-standardized). H^2^, broad-sense heritability; Ve, error variance component; Vg, genetic variance component; Noutliers, number of outliers. Analysis based on resampling of 5, 10, 20, 30, or 40 roots from each plot at random.

**Figure 7:**
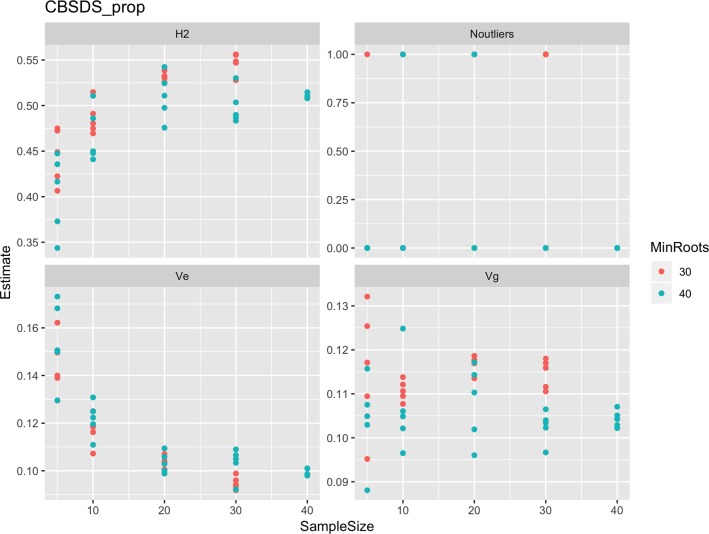
Influence of root sample sizes on proportion-based root necrosis index (CBSD-proportion). H^2^, broad-sense heritability; Ve, error variance component; Vg, genetic variance component; Noutliers, number of outliers. Analysis based on resampling of 5, 10, 20, 30, or 40 roots from each plot at random.

Roots assessed per plot were associated with high and variable standard errors (**Figure 1**). Thus, to empirically establish whether or not roots assessed per plot had an influence on overall CBSD ranking of cassava clones, we examined seven categories of root samples. We observed that root sample size significantly affected CBSD root necrosis assessment methods (**Table 4**). For example, significant differences in CBSDRs were only observed between root samples sizes: 21 and 30 roots, 41 and 50 roots, and between 51 and 60 roots. On the other hand, we only observed significant differences in CBSDRi between root sample sizes of 51 and 60 roots (**Table 4**). For both CBSD-Harmonic and CBSD-standardized, significant differences were observed across all root sample sizes except sample size of 71–96 roots (**Table 4**). No significant differences for CBSD-proportion were observed across all root sample sizes. When sub sampling of data is undertaken, (minimum number of roots/plot being 30 or 40), similar patterns are observed (**Figures 4**–**7**).

**Table 4:** Influence of root sample size on CBSD root necrosis assessment.

Sampled Roots	CBSDRs			CBSD− Harmonic			CBSD− Proportion			CBSD− Standardized			CBSD− Proportion		
	Est.	SE	p.value	Est.	SE	p.value	Est.	SE	p.value	Est.	SE	p.value	Est.	SE	p.value
11–20	– 0.09	0.06	0.140	– 0.62	2.14	0.772	– 0.18	0.05	0.002	– 0.61	0.17	0.000	– 0.05	0.04	0.222
21–30	– 0.17	0.07	0.023	– 4.16	2.53	0.100	– 0.22	0.07	0.001	– 0.71	0.20	0.001	– 0.01	0.05	0.773
31–40	– 0.09	0.08	0.265	– 2.21	3.03	0.466	– 0.19	0.08	0.022	– 1.03	0.24	0.000	– 0.01	0.06	0.820
41–50	– 0.24	0.09	0.009	– 5.20	3.14	0.097	– 0.27	0.08	0.001	– 0.96	0.25	0.000	0.01	0.06	0.865
51–60	– 0.34	0.11	0.003	– 7.81	3.91	0.046	– 0.38	0.10	0.000	– 1.02	0.31	0.001	0.03	0.08	0.689
61–70	– 0.16	0.15	0.301	– 3.55	5.32	0.504	– 0.30	0.14	0.039	– 1.78	0.43	0.000	– 0.02	0.11	0.848
71–96	– 0.26	0.17	0.121	– 9.04	5.87	0.123	– 0.28	0.16	0.078	0.11	0.48	0.811	0.11	0.12	0.373

CBSDRi, cassava brown streak disease root incidence; CBSDRs, cassava brown streak disease root severity; CBSD-Harmonic, cassava brown streak disease root severity computed as harmonic mean; CBSD-proportion, proportion-based root necrosis index; CBSD-standardized, standardized root necrosis index. Est, estimate; SE, standard error; p-value, probability for significance tested at 5%.

## Discussion

So often, concepts applied for plant disease assessment of foliar parts suffice for roots (Kranz, 1988; Lee and Madden, 1990). However, CBSVs present a unique situation such that upon infection, characteristic CBSD symptoms are manifested on leaves, stems and roots, with consistently poor correlations of disease severities and/or incidences amongst foliar and roots (Hillocks and Jennings, 2003; Kaweesi et al., 2014; Okul Valentor et al., 2018). The starch-bearing roots, which by far are the most economically important part of cassava, are worst hit by CBSD. This was the motivation for this study.

Principally, our motivation was driven by two fundamental challenges that are routinely encountered by CBSD resistance breeding programs. Firstly, the fact that different genotypes exhibit varying frequencies of root severity scores per plant, and that the number of roots assessed per plant and/or per plot vary considerably. Secondly, that a root scored as either 1 or 2 is economically more valuable than a root that is assigned a score of 3, 4, or 5. Thus, economic value of roots takes the order of 1 > 2 > 3 > 4 > 5. These insights have never been considered in ranking cassava clones, and yet they have a huge bearing on *per se* performance of cassava clones during CBSD resistance screening. Thus, herein we compared traditional and unweighted CBSD root necrosis assessment methods (CBSDRi and CBSDRs, CBSD-Harmonic) with CBSD root necrosis indexes (CBSD-standardized and CBSD-proportion), which account for the variable number of roots assessed/plot. These assessment methods can be equated to traits to guide the selection process.

Principally, disease assessment methods should be accurate, reproducible and economical (Lee and Madden, 1990). This is particularly important in the current era where breeding programs experience acute economic constraints and thus, need to optimize their operations. Thus, we computed standard errors associated with each unit of the 1–5 CBSD root severity scale (**Figure 1**) and assessment methods (**Figure 2**). Higher and more variations in standard errors were consistently associated with severity score 1; other severity scale levels (particularly 2, 3, and 4) were associated with lower and less variable standard errors. Amongst the five CBSD root necrosis traits, highest and more variation in standard errors were associated with CBSDRi (**Table 1**; **Figure 2**). Among the evaluation sites, Namulonge was consistently associated with lower standard errors.

High variability of standard errors associated with severity score 1 illustrates the high variability in number roots of a specific clone scored 1 across seasons and/or locations. This is most likely a function of differences in virus inoculum, strain aggressiveness and/or species diversity among evaluation sites and/or seasons. On the other hand, the low standard errors associated with the Namulonge dataset are indicative of less skewed distributions of data points arising from the 1–5 scale and/or the size of the sampling units (*number of roots assessed per plot)*. This finding qualifies Namulonge as an appropriate site for undertaking CBSD resistance screening, as demonstrated by other studies (Kaweesi et al., 2014; Kawuki et al., 2016; Okul Valentor et al., 2018). Indeed, Namulonge, located in central Uganda, could be considered as a national, regional or international site for screening cassava for CBSD resistance. The combination of mixed CBSV strains and high CBSD pressure at Namulonge has been recognized by the Next Generation Cassava Breeding Project (http://www.nextgencassava.org), which currently undertakes CBSD field resistance screening of Latin American and West African cassava germplasm at Namulonge.

The five CBSD root necrosis assessment methods (traits) namely CBSDRi, CBSDRs, CBSD-Harmonic, CBSD-proportion, and CBSD-standardized, had their respective estimated plot-based heritabilities of 0.44, 0.49, 0.44, 0.79, and 0.22 (**Table 1**). Heritability, an important concept in selective breeding, provides population-specific information on what proportion of phenotypic variation is genetic. Previous studies conducted in Uganda have only estimated heritabilities for CBSDRi and CBSDRs, whose estimates respectively range from 0.37 to 0.5 (Ozimati et al., 2018), and/or from 0.25 to 0.64 (Okul Valentor et al., 2018). Thus, CBSDRs and CBSDRi heritabilities estimated from the current study are comparable to those from previous studies. Of keen interest from this study is the high heritability for CBSD-proportion index (0.79), which is negatively correlated to both CBSDRi (r = −0.76) and CBSDRs (r = −0.66). This result suggests that CBSD-proportion index is a more repeatable method only that the values that it uses to categorize clones are opposite to values used by traditional CBSD root necrosis assessment methods, CBSDRi and CBSDRs. Indeed, for this index, the more positive the value, the higher the respective clone resistance, and the more negative the value, the more the respective clone CBSD susceptibility.

In our endeavor to systematically increase genetic gain for key cassava traits, notable of which is CBSD resistance, adoption of accurate evaluation and selection criteria is critical. It also suffices to note that disease assessments are often done for several reasons: quantifying effects on yield or disease distribution, testing efficacies of plant protection chemicals, forecasting disease outbreaks, and in variety selection trials (Lee and Madden, 1990). Within limits, datasets presented in this study justify the use of four disease assessment methods (CBSDRi, CBSDRs, CBSD-Harmonic, and CBSD-proportion) for variety selection trials, as they were associated with moderate to high heritabilities i.e., H^2^ > 0.4. With the use of electronic data collection tools and the availability of databases, e.g. cassavabase (www.cassavabase.org), the four disease assessments methods (traits) should be applied to increase on the rigor of CBSD necrosis assessment and thus contributing truly to the attainment of genetic gain to either be deployed in farmers’ fields or to be used in crossing nurseries.

To further assess the accuracy of CBSD-related traits in discriminating cassava clones, we compared how each assessment method could rank clones based on computed BLUPs (**Table 3**). BLUPs offer an excellent predictive accuracy to guide selection (Panter and Allen, 1995; Piepho et al., 2008). Indeed, one clone (UG130014) featured in each of the five CBSD root necrosis assessment traits, while five clones (UG120180, UG120063, UG130002, UG130033, and UG120183) featured with three (CBSD-Harmonic, CBSDRi, and CBSDRs).

Highly susceptible clones were readily discernible by the five CBSD root necrosis-related traits (**Table 3**). Clone UG130014 had an average of 27.4 roots assessed, with average CBSDRi, CBSDRs, CBSD-proportion, and CBSD-standardized of 1.45%, 1.01, 0.97, and 2.13, respectively; clone UG120156 had an average of 31.2 roots assessed, with average CBSDRi, CBSDRs, CBSD-proportion, and CBSD-standardized of 4.4%, 1.04, 0.91, and 1.98; clone UG120063 had an average of 10.6 roots assessed, with average CBSDRi, CBSDRs, CBSD-proportion, and CBSD-standardized of 4.89%, 1.06, 0.86, and 0.88 respectively. The highly susceptible clone UG110016 had an average of 20.7 roots assessed, with average CBSDRi, CBSDRs, CBSD-proportion, and CBSD-standardized of 92.2%, 4.27, −0.32, and −1.28, respectively (**Supplementary Table 1**). Basing on the BLUP rankings, it’s evident that resistance grouping, as expected was variable (owing to whether or not sample size or weights are included in evaluations), while susceptible grouping tended to be similar.

It is therefore apparent that index-based assessment methods (CBSD-standardized and CBSD-proportion) provide an opportunity for extracting useful information from root necrosis data by factoring in the variable root sample size to inform selection decisions. Accordingly, clones with higher positive values merit for selection and/or advancement. Practically, use of this index is appropriate for early selection stages clonal trials that are often characterized by several entries with highly variable number of roots, the key sampling unit for CBSD root necrosis assessment. In situations where late selection stages i.e., advanced or uniform yield trials, exhibit varying roots per plot, these indexes could also be used to inform selection decisions.

Effectiveness of resistance screening hinges on balancing statistical, biological and/or economic considerations (Neher and Campbell, 1994). Roots assessed per plot were associated with high and variable standard errors (**Figure 1**). Thus, to underpin the influence of root sample sizes on CBSD root necrosis assessment, we examined seven categories of root samples from which we observed that root sample size, does indeed have a significant effect on CBSD root necrosis assessment (**Table 4**). In fact, the importance of root sample size can be illustrated when we compared data for three contrasting clones i.e., UG120156, UG120063, and UG110016. These clones were compared using an average of 31.2, 10.6, and 20.7 roots assessed per clone, respectively (**Supplementary Table 1**). The respective CBSDRi, CBSDRs, CBSD-proportion, and CBSD-standardized associated with these clones were: 4.43%, 1.04, 0.91, and 1.98, for UG120156; 4.9%, 1.06, 0.86, and 0.88, for UG120063; and 92.2%, 4.27, −0.32, and −1.28, for UG110016. Clearly, this variable number of roots assessed per clone, in addition to the differences in root severity classes further justify the use of indexes particularly root numbers vary considerably among plots. With increase in samples sizes i.e., in advanced or uniform yield trials, then use of CBSDRi and/or CBSDRs should predominant over the indices CBSD-proportion and/or CBSD-standardized.

Utility of virus titre from leaf, stem or root tissues to guide selection has been limited due to several logistical and/or biological constraints. Herein, we have generated information that could be used to improve CBSD resistance screening and thus optimize breeding operations. Consequently, three conclusions are apparent. First, with the exception of CBSD-standardized, all other evaluated CBSD root necrosis assessment methods (CBSDRi, CBSDRs, CBSD-Harmonic, and CBSD-proportion) had moderate to high heritability, and can thus be used for CBSD root necrosis evaluations. Correlations associated between assessment methods, despite direction, reinforce this conclusion. Second, low standard errors associated with Namulonge (NaCRRI), qualify it as an appropriate site for undertaking stage-gate selections for CBSD particularly in early selection stages. Third, the significant influence of root sample size on overall ranking of clones justify the use of CBSD root necrosis indexes in early selection stages i.e., clonal trials, that are often characterized by several entries with highly variable number of roots, the key sampling unit for CBSD root necrosis assessment.

## Data Availability Statement

All datasets generated for this study are included in the article/**Supplementary Material**.

## Author Contributions

WE: involved in data collection and write-up. AO: involved in data collection and data analysis. IK: involved in data collection and manuscript writing. LN: involved in manuscript writing. MW: involved in data analysis and manuscript editing. RK: was involved in data collection, analysis and manuscript writing.

## Funding

Funding for this work was provided by Cornell University through a sub-award agreement (N0. 84941-11038) between NaCRRI and Cornell University

## Conflict of Interest

The authors declare that the research was conducted in the absence of any commercial or financial relationships that could be construed as a potential conflict of interest.
